# MiR-544 promotes immune escape through downregulation of NCR1/NKp46 via targeting RUNX3 in liver cancer

**DOI:** 10.1186/s12935-018-0542-y

**Published:** 2018-04-03

**Authors:** Chenwei Pan, Luxia Xiang, Zhenzhen Pan, Xiaodong Wang, Jie Li, Lu Zhuge, Peipei Fang, Qipeng Xie, Xuezhen Hu

**Affiliations:** 1 Department of Infectious Disease, The Second Affiliated Hospital and Yuying Children’s Hospital of Wenzhou Medical University, No. 109 West College Road, Wenzhou, 325027 Zhejiang China; 2 The Second School of Medicine, Wenzhou Medical University, Wenzhou, 325000 China; 3 Department of Infectious Disease, The First Affiliated Hospital of Wenzhou Medical University, Wenzhou, 325000 China; 4 Department of Emergency Medicine, The Second Affiliated Hospital and Yuying Children’s Hospital of Wenzhou Medical University, Wenzhou, 325027 China

**Keywords:** miR-544, RUNX3, NCR1, NK cells, Immune escape

## Abstract

**Objective:**

To study the potential role of miR-544 in the immune escape mechanism of hepatoma cells.

**Methods:**

Natural killer (NK) cells were collected from healthy volunteers and patients with liver cancer. Interleukin (IL)-2 activated-NK-92 cells were transfected with miR-544 inhibitor/mimic or NC/pre-NC in HepG2 co-culture system. NK-92 cells were treated with control, IL-2, IL-2 + pre-NC, IL-2 + miR-544 mimic, IL-2 + miR-544 mimic + pcDNA and IL-2 + miR-544 mimic + pcDNA-runt-related transcription factor 3 (RUNX3) groups. Mice models of liver cancer were well established. Expression of miR-544, natural cytotoxicity receptor 1 (NCR1) and RUNX3 were evaluated by quantitative real-time PCR and western blotting. Flow cytometry and ELISA were used to determine NK cell cytotoxicity and the levels of INF-γ, respectively.

**Results:**

MiR-544 was upregulated while NCR1 and RUNX3 was downregulated in NK cells of patients with liver cancer. The levels of IFN-γ and miR-544 expression were increased and decreased in IL-2 activated-NK cells, respectively. Inversely, miR-544 overexpression inhibited NK cell cytotoxicity by downregulating IFN-γ. However, miR-544 directly targeted RUNX3 and negatively regulated NCR1. Furthermore, miR-544 promoted immune escape of hepatoma cells in vivo and in vitro.

**Conclusion:**

miR-544 promoted the immune escape of liver cancer cells by downregulating NCR1 via targeting RUNX3.

## Background

Primary liver cancer (PLC) remains the fifth most common malignancy that accounted for an estimated 746,000 new deaths each year worldwide, ranking third among the overall cause of death from tumor [1, 2]. Accumulating reports have provided evidence that PLC usually concealed onset with nonspecific clinical manifestation in the early stage [3]. Generally, clinical symptoms were present in the intermediate and advanced stage. At present, operative treatment combined with adjuvant interventional therapy, chemoembolization and target biotherapy were the major therapeutic strategies, however, surgical resection and liver transplantation were the main radical cures which always led to more complications with high risk of recurrence [4–7]. Thus, there was an urgent need to develop a novel therapy for relapse prevention. Increasing evidence has demonstrated that immunotherapy for cancer played a potential role in destroying malignant cells through activating anti-tumor immune responses or adoptively transfusing tumor infiltrating lymphocytes (TIL) [8]. Nonetheless, the overall curative outcome was considered unsatisfactory on account of the immunotolerance mechanism in tumor escaping from immunological surveillance which was defined as the immune escape [9]. Whereas, the specific molecular mechanism involving immunotolerance remains unclear [10].

The natural immune system of liver was greatly distinct from other tissues or organs, comprising a great deal of resident innate immune cells containing macrophages, NK cells and NK-T (NKT) cells. Particularly, NK cells had the capability of reacting directly to dangerous signals resulting in eliminating abnormal cells including pathogenic microorganisms-infected cells and malignant cells. Therefore, NK cells acted as the first line of defense against cancer and infection [11]. However, dysregulated expression of NK cell activating, inhibitory receptors and their ligands impaired the function of NK cells in tumor microenvironment, inducing immune tolerance and dysfunction which eventually led to immune escape. Consequently, immunotherapy based on the reversion of the imbalance of receptors and corresponding ligands expression might represent an attractive approach for patients with PLC [12].

NKp46 encoded by NCR1 was identified as a pivotal member of NCR family which was specifically expressed on both resting and activated NK cells, acting as a tumor suppressor in tumor growth and metastasis [13]. Although it has been testified that decreased NKp46 expression and dysfunction of NK cells were found in various solid tumors and hematological malignancies [14, 15]. Nevertheless, abnormal expression of NKp46 and its involvement in tumor immune escape mechanism were not yet been confirmed. Additionally, Lai et al. illustrated the role of RUNX3 in modulating transcription regulation of NCR1 [16].

Recently, a number of microRNAs (miRNAs) have been reported as crucial regulators of managing immune cell development and function such as miR-29 [17], miR-15/16 [18] and miR-25-93-106b cluster [19]. More recently, Qiu et al. reported that miR-544 was associated with the expression of RUNX3 as well as a series of cytokines, such as IL-2, IL-4, IL-10 and IFN-γ in T helper cell immune responses [20].

In this study, we thus explored the underlying role of miR-544 in the mechanism of tumor immune escape, with an eye toward developing a promising novel approach for improving NK cell-mediated immunotherapy to treat liver cancer.

## Materials and methods

### Isolation and culture of primary human NK cells

Peripheral blood mononuclear cells (PBMCs) were isolated from venous blood obtained from healthy adult volunteers and patients with liver cancer (n = 120 per group) in The Second Affiliated Hospital and Yuying Children’s Hospital of Wenzhou Medical University (Wenzhou, Zhejiang, China) who signed informed consent by using a Ficoll-Hypaque (Pharmacia Biotech, Uppsala, Sweden) density gradient centrifugation technique and were re-suspended in Roswell Park Memorial Institute (RPMI) 1640 medium (Invitrogen, Camarillo, CA, USA) supplemented with 10% fetal bovine serum (FBS) (Sigma-Aldrich, St. Louis, MO, USA). NK cells were harvested by the negative selection procedure of magnetic activated cell sorting (MACS) using an Human NK cells separation medium kit (Sangon Biotech, Shanghai, China) according to the manufacturer’s instructions and purified by differential attachment using flow cytometry. The qRT-PCR analysis and western blot were used to detect the mRNA expression levels of miR-544 and NCR1 and RUNX3 expression at protein levels.

### Cell treatment

Human NK cell lines NK-92 purchased from American Type Culture Collection (ATCC, Manassas, VA, USA) were stimulated with 20 ng/mL of IL-2 (BD Biosciences, San Diego, CA, USA) for 24 h or cultured in RPMI-1640 medium supplemented with 10% FBS as a control. The cells were collected for determining miR-544 mRNA expression levels and the levels of INF-γ levels using ELISA.

### NK-92 cells transfection and co-culture with HepG2 cells

1 × 10^5^/mL of human hepatic cancer cell line HepG2 (ATCC) diluted with RPMI-1640 medium supplemented with 10% FBS were seeded in 96-well plates at 100 μL/well. Afterwards, IL-2-stimulated NK-92 cells were transfected with miR-544 mimic/inhibitor or pre-NC/NC (Shanghai GenePharma Co., Ltd., Shanghai, China) as controls, followed by co-cultured with HepG2 at effector cell/target cell (E:T) ratios of 10:1 for 4 h at 37 °C with 5% CO_2_. After centrifugation, the supernatants were harvested to measure the levels of INF-γ. In addition, NK cells were harvested for testing the expression of miR-544 at mRNA levels as well as cytotoxicity using flow cytometry.

### Luciferase reporter assay

The 3′-UTR fragments of RUNX3 and the potential target sequences of miR-544 predicted by Targetscan (http://www.targetscan.org) were amplified and sub-cloned into pISo plasmids (Promega, Madison, WI, USA). 293T cells (American Type Culture Collection, Manassas, VA, USA) grown to about 70% confidence were seeded in 24-well plates and then co-transfected with RUNX3-WT vectors or RUNX3-MUT vectors (50 ng) along with miR-544 mimic/inhibitor or their corresponding empty vectors (pre-NC/NC) using Lipofectamine 2000 (Invitrogen). Luciferase activity was measured 24 h after transfection using a Dual-Luciferase Reporter 1000 Assay System (Promega) according to the manufacturer’s instructions. To further verify the role of miR-544 in regulating RUNX3 in NK cells, the relative expression of RUNX3 at mRNA and protein levels following transfection of miR-544 inhibitor or mimic into NK-92 cells were measured by qRT-PCR and western blotting, respectively. Besides, the miR-544 mRNA expression levels in N-92 cells following transfection of miR-544 mimic or pre-NC were determined.

### Cell transfection

NK-92 cells were divided into the control, IL-2, IL-2 + pre-NC, IL-2 + miR-544 mimic, IL-2 + miR-544 mimic + pcDNA and IL-2 + miR-544 mimic + pcDNA-RUNX3 groups. Before 24 h of transfection, NK-92 cells were primarily cultured, passaged thrice and seeded in 6-well plates. After the confluence reached 30–50%, cell transfection was performed using Lipofectamine 2000 reagent (Invitrogen) following the manufacturer’s instructions. 24 h later, the medium was replaced with fresh RPMI-1640 media and cultured at 37 °C with 5% CO_2_. After 72 h of transfection, cells were collected for detecting miR-544 mRNA expression levels, RUNX3 protein expression, NCR1 mRNA expression and the percent of NKp46^+^NK cells. Besides, cells in the control group were cultured in serum-free medium.

In addition, aforementioned NK-92 cells following transfection were co-cultured with HepG2 cells, and the levels of INF-γ as well as cytotoxicity were determined.

### Nude mice xenograft model

Mice model of liver cancer was well established as previously described [21]. Briefly, HepG2 cells were transferred into the armpit of the right forelimb of male BALB/c-nude mice (7 week old, weighing 18–20 g) which were purchased from the Animal Center of Wenzhou Medical University. On the 5th day after mice model was successfully constructed, IL-2 stimulated-mice NK cell line LNK was transfected with pre-NC and miR-544 mimic and subcutaneously injected into the tumor sites of 6 mice per group at a density of 6 × 10^7^ cells/mL (50 μL) for 5 consecutive days. Tumor volume was measured every 3 days by the formula: Tumor volume (cm^3^) = length × width^2^ × 0.52. After 12 days, the mice in each group were sacrificed. Tumor tissues were separated and the mRNA expression of miR-544 and NCR1 and the protein expression of RUNX3 were detected by RT-PCR and western blot analysis. All animal protocols were approved by Ethics Committee of The Second Affiliated Hospital and Yuying Children’s Hospital of Wenzhou Medical University.

### Quantitative real-time PCR

Total RNA was extracted from NK cells obtained from healthy volunteers and patients with liver cancer and NK-92 cell lines following transfection as well as tumor tissues using Trizol reagent (Invitrogen) and then reverse-transcribed to cDNA using a TaqMan MicroRNA Reverse Transcription kit (Applied Biosystems, Carlsbad, CA, USA) according to the manufacturer’s protocol. Quantitative real-time PCR (qRT-PCR) was performed on an ABI 7500 Real-Time PCR system (Applied Biosystems) using TaqMan Gene Expression Master Mix (Applied Biosystems). Relative quantification in triplicate of miRNA-544 and NCR1 expression was calculated using the 2^−∆∆CT^ method. The relative expression of mRNAs and miRNAs were normalized to β-actin and U6 snRNA expression, respectively.

The specific primers used were as follows: miR-544: 5′-GGAACTCGAGCCGCTGCCTTAAGTCACTCTCTAT-3′ and 5′-GGAAGGATCCCACAACTGACCACAGTTT-3′; NCR1: 5′-TCTAGACGGCAGTAGAAGGTC-3′ and 5′-CTTGCTGGATCTGGTGGTAA-3′.

### Western blot

Total protein extraction of NK cells, NK-92 cells lines and tumor tissues was performed using RIPA lysis buffer (Beijing Solarbio Science and Technology, Beijing, China). Protein samples were separated with 10% sodium dodecyl sulfate polyacrylamide gel electrophoresis (SDS-PAGE), transferred onto polyvinylidene fluoride (PVDF) membranes (Millipore) and blocked with tris buffered saline tween (TBST) buffer containing 5% non-fat milk at room temperature for 1 h. The membranes were incubated with primary antibodies rabbit anti-RUNX3 (1:1000; Cell Signaling Technology, Boston, MA, USA) and anti-NKp46 (1:1000; Bioworld Technology Inc. Minneapolis, MN, USA) at 4 °C overnight, followed by rinsed with TBST thrice, and then incubated with goat anti-rabbit IgG (Zhongshan Golden Bridge Biotechnology, Beijing, China) at room temperature for 1 h under a shaker. Protein bands were visualized using an enhanced chemiluminescence reagent (Beckman Coulter, Brea, CA, USA). Band intensities were standardized to β-actin (Sigma-Aldrich) as a loading control.

### ELISA detection of INF-γ

For the detection of INF-γ levels, the cells were washed with PBS, cultured in culture medium and seeded into 96-well plates at a density of 5 × 10^3^ cells/well for 4 h at 37 °C with 5% CO_2_, followed by centrifugation at 500 rpm for 4 min. Supernatants were collected to detect the levels of IFN-γ using IFN-γ Human ELISA Kit (Invitrogen) following manufacturer’s protocols. Optical density (OD) was measured at 450 nm with an ELISA reader (Bio-Rad; Hercules, CA, USA).

### Cytotoxicity assay

NK cells cytotoxicity against hepatic cancer cells was analyzed using flow cytometry assay. HepG2 target cells were stained with 5 mg/mL calcein acetoxymethyl ester (CAM; Sigma-Aldrich) for 1 h at 37 °C, washed once with PBS and cultured in 96-well plates with RPMI-1640 medium, followed by measured total cytotoxicity after the addition of 2.5% Triton X-100 for 30 min. The target cells and effector cells (NK-92 cells) following transfection were co-cultured at the indicated E:T ratios, incubated for 4 h at 37 °C in a humidified incubator containing 5% CO_2_ and centrifuged at 500 rpm for 5 min. The supernatants were removed and PBS was added in each well. The percentage of NK cell cytotoxicity was calculated using FACSCalibur system (BD Biosciences).

### Flow cytometry analysis of NKp46^+^ in NK cells

NK-92 cells following 72 h transfection were harvested, washed with PBS and incubated with PE-labeled anti-NKp46 (BD Biosciences) for 30 min at 4 °C. After rinsed with PBS twice, cells were then incubated with FITC-labeled goat anti-mouse IgG (BD Biosciences) for another 30 min at 4 °C in the dark, and analyzed by flow cytometry using a FACSCalibur flow cytometer (BD Biosciences).

### Statistical analysis

All data were presented as the mean ± standard deviation (SD). Statistical analyses were performed with SPSS version 18.0 software. Two-sided Student’s t test was used to analyze the differences between groups, and a P value < 0.05 was considered significant.

## Results

### MiR-544 was upregulated while NCR1 and RUNX3 was downregulated in NK cells of patients with liver cancer

**Fig. 1:**
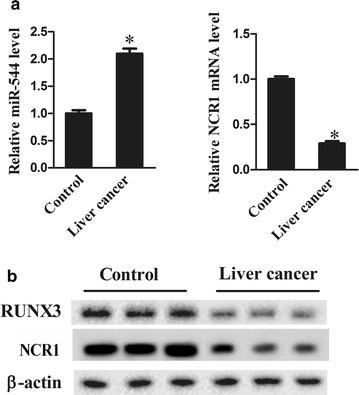
MiR-544, NCR1 and RUNX3 expression in NK cells. **a** The relative mRNA expression of miR-544 and NCR1 in NK cells obtained from healthy volunteers and patients with liver cancer using qRT-PCR. **b** The protein expression levels of RUNX3 and NCR1 in NK cells obtained from healthy volunteers and patients with liver cancer using western blotting. *Refers to compare with the control group, P < 0.05

The relative mRNA expression levels of miR-544 and NCR1 as well as the expression of RUNX3 at protein levels were measured using qRT-PCR and western blotting in NK cells isolated from healthy volunteers and patients with liver cancer. MiR-544 was significantly upregulated (Fig. 1a; P < 0.05) while NCR1 (Fig. 1a, b; P < 0.05) and RUNX3 (Fig. 1b; P < 0.05) were markedly downregulated in NK cells of liver cancer patients compared with those of healthy population, hinting that miR-544 level was negatively associated with the expression of NCR1 and RUNX3.

### IL-2 activated NK cells, inhibited secretion of IFN-γ and downregulated miR-544

**Fig. 2:**
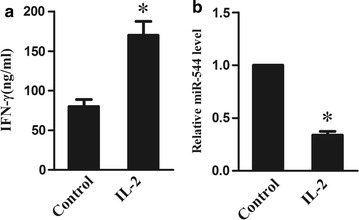
Effect of IL-2 on IFN-γ and miR-544 expression in NK cells. **a** The levels of IFN-γ in NK-92 cells with or without IL-2 induction using ELISA. **b** The relative mRNA expression of miR-544 in NK-92 cells with or without IL-2 induction. *Refers to compare with the control group, P < 0.05

Next, we evaluated the effect of IL-2 on mediating miR-544 in vitro. Human NK-92 cells showed the elevated levels of IFN-γ (Fig. 2a; P < 0.05) and the decreased levels of miR-544 expression (Fig. 2b; P < 0.05) in response to IL-2, suggesting NK cells could be activated by IL-2, leading to the increased secretion of IFN-γ and the suppressive expression of miR-544.

### MiR-544 attenuated NK cell-mediated cytotoxicity by downregulating IFN-γ

**Fig. 3:**
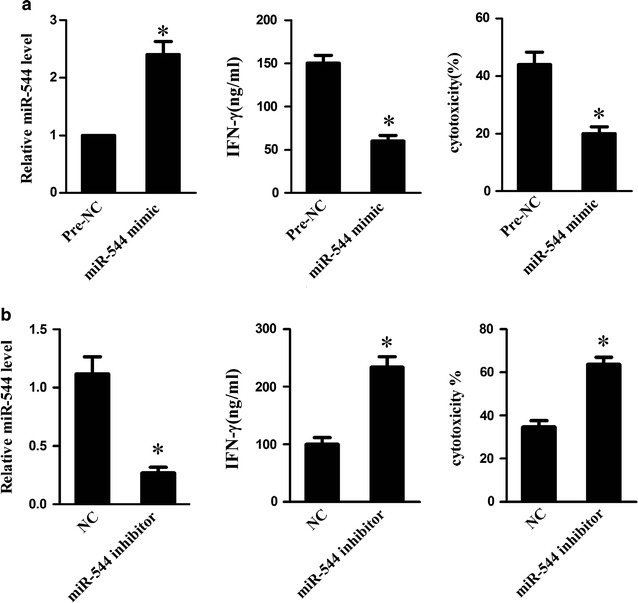
Effect of miR-544 on IFN-γ and NK cells cytotoxicity. **a** The relative mRNA expression of miR-544 in IL-2 activated-NK-92 cells following transfection with pre-NC or miR-544 mimic. The levels of IFN-γ in IL-2 activated-NK-92 cells following transfection with pre-NC or miR-544 mimic. The cytotoxicity of IL-2 activated-NK-92 cells following transfection with pre-NC or miR-544 mimic using flow cytometry. **b** The relative mRNA expression of miR-544 in IL-2 activated-NK-92 cells following transfection with NC or miR-544 inhibitor. The levels of IFN-γ in IL-2 activated-NK-92 cells following transfection with NC or miR-544 inhibitor. The cytotoxicity of IL-2 activated-NK-92 cells following transfection with NC or miR-544 inhibitor using flow cytometry. *Refers to compare with the pre-NC group, P < 0.05

We then investigated the role of miR-544 in modulating cell cytotoxicity of NK cells. The results demonstrated that miR-544 overexpression observably upregulated miR-544 in co-culture of HepG2 cells and NK-92 cells during IL-2 induction, alleviated IFN-γ and reduced the cytotoxicity of NK-92 cells against HepG2 cells (Fig. 3a; P < 0.05), whereas, miR-544 knockdown led to an opposite effect (Fig. 3b; P < 0.05). These findings implied that miR-544 suppressed the killing effect of NK cells on human hepatic cancer cells through diminishing IFN-γ secretion.

### MiR-544 downregulated NCR1 by targeting RUNX3

**Fig. 4:**
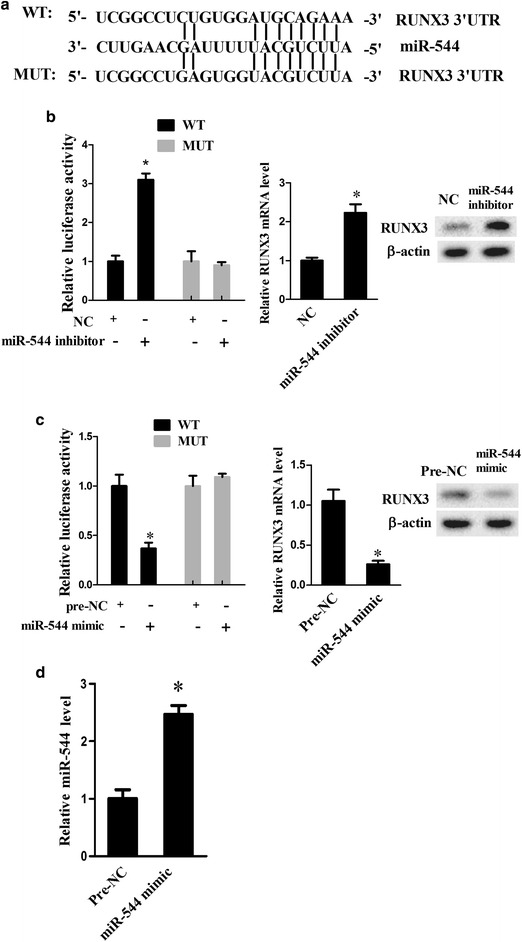
The negative RUNX3 regulation by miR-544. **a** The potential binding sites of miR-544 and RUNX3 predicted by bioinformatical analysis. **b** The relative luciferase activity in NK-92 co-transfected with miR-544 inhibitor or NC together with WT or Mut vector plasmids of RUNX3. The expression of RUNX3 at mRNA and protein levels in NK-92 cells transfected with miR-544 inhibitor or NC. *Refers to compare with the NC group, P < 0.05. **c** The relative luciferase activity in NK-92 co-transfected with miR-544 mimic or pre-NC together with WT or Mut vector plasmids of RUNX3. The expression of RUNX3 at mRNA and protein levels in NK-92 cells transfected with miR-544 mimic or pre-NC. **d** The mRNA expression levels of miR-544 in NK-92 cells co-transfected with miR-544 mimic or pre-NC. *Refers to compare with the pre-NC group, P < 0.05

Bioinformatics analysis used for predicting recognition sequences on RUNX3 revealed the presence of miR-544 binding site (Fig. 4a). We examined whether RUN3 was a direct target gene of miR-544 using Luciferase reporter assay. The results showed that miR-544 inhibitor resulted in an enhancement of luciferase activity in comparison with the NC group (Fig. 4b, P < 0.05), whereas, miR-544 mimic led to an opposite effect relative to the pre-NC group (Fig. 4c, P < 0.05) in RUNX3-WT group. However, neither miR-15b mimic nor inhibitor notably affected luciferase activity in the RUNX3-Mut group. Furthermore, miR-544 knockdown ascended the expression of RUNX3 at mRNA and protein levels (Fig. 4b; P < 0.05). In contrast, miR-544 overexpression negatively regulated RUNX3 at mRNA and protein levels (Fig. 4c; P < 0.05), since miR-544 overexpression upregulated miR-544 mRNA expression (Fig. 4d; P < 0.05). Collectively, miR-544 negatively regulated RUNX3 expression.

**Fig. 5:**
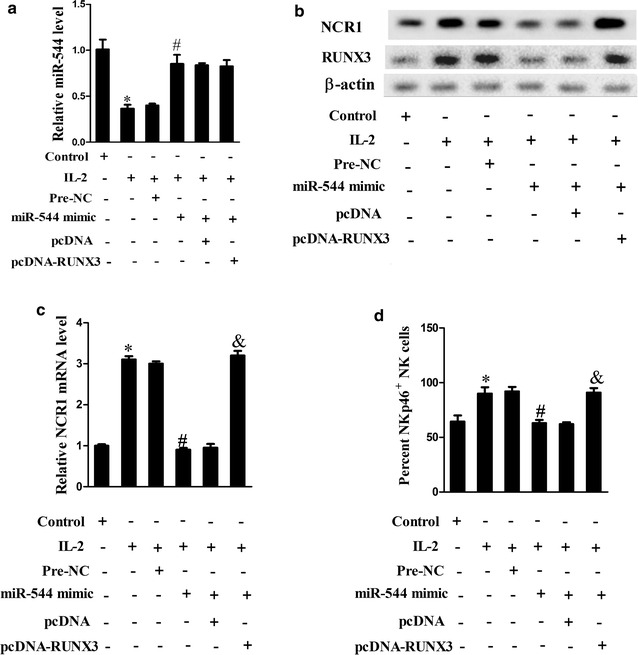
Effect of miR-544 on NCR1 expression. **a** The mRNA expression levels of miR-544 in control, IL-2, IL-2 + pre-NC, IL-2 + miR-544 mimic, IL-2 + miR-544 mimic + pcDNA and IL-2 + miR-544 mimic + pcDNA-RUNX3 groups. **b** The protein expression levels of NCR1 and RUNX3 in control, IL-2, IL-2 + pre-NC, IL-2 + miR-544 mimic, IL-2 + miR-544 mimic + pcDNA and IL-2 + miR-544 mimic + pcDNA-RUNX3 groups. **c** The mRNA expression of NCR1 in control, IL-2, IL-2 + pre-NC, IL-2 + miR-544 mimic, IL-2 + miR-544 mimic + pcDNA and IL-2 + miR-544 mimic + pcDNA-RUNX3 groups. **d** The percent of NKp46 + NK cells in control, IL-2, IL-2 + pre-NC, IL-2 + miR-544 mimic, IL-2 + miR-544 mimic + pcDNA and IL-2 + miR-544 mimic + pcDNA-RUNX3 groups using flow cytometry. *Refers to compare with the control group, P < 0.05. ^#^Refers to compare with the IL-2 + pre-NC group, P < 0.05. ^&^Refers to compare with the IL-2 + miR-544 mimic + pcDNA group, P < 0.05

NK-92 cells induced with IL-2 downregulated the mRNA levels of miR-544 (Fig. 5a; P < 0.05), elevated the protein expression of NCR1 and RUNX3 (Fig. 5b; P < 0.05), NCR1 mRNA expression (Fig. 5c; P < 0.05) and the percentage of NKp46^+^NK cells (Fig. 5d; P < 0.05), while miR-544 overexpression had the opposite trends as compared with the IL-2 + pre-NC group (all P < 0.05). Nevertheless, simultaneous overexpressing miR-544 and RUNX3 reversed the effect of miR-544 overexpression in comparison with the IL-2 + miR-544 mimic + pcDNA group (all P < 0.05), indicating that miR-544 overexpression could negatively regulated NCR1 by regulating RUNX3.

### MiR-544 facilitated immune escape of hepatoma cells by downregulating RUNX3

**Fig. 6:**
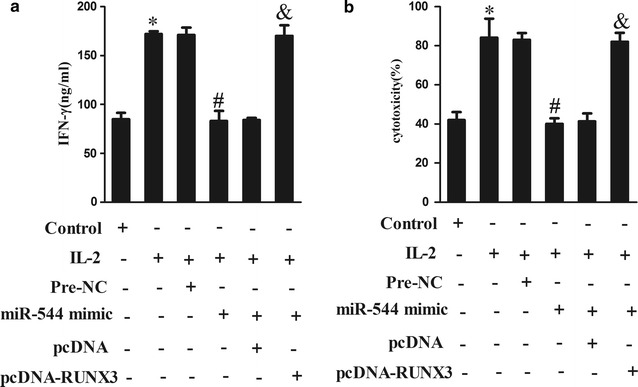
Effect of miR-544 on immune escape of hepatoma cells in vitro. **a** The levels of IFN-γ in control, IL-2, IL-2 + pre-NC, IL-2 + miR-544 mimic, IL-2 + miR-544 mimic + pcDNA and IL-2 + miR-544 mimic + pcDNA-RUNX3 groups. **b** The cytotoxicity of NK-92 cells in control, IL-2, IL-2 + pre-NC, IL-2 + miR-544 mimic, IL-2 + miR-544 mimic + pcDNA and IL-2 + miR-544 mimic + pcDNA-RUNX3 groups. *Refers to compare with the control group, P < 0.05. ^#^Refers to compare with the IL-2 + pre-NC group, P < 0.05. ^&^Refers to compare with the IL-2 + miR-544 mimic + pcDNA group, P < 0.05

To explore the underlying role of miR-544 in regulating immune escape mechanism of liver cancer, the levels of IFN-γ and cytotoxicity of NK-92 cells following transfection in HepG2 cells co-culture system were measure. The results displayed that IFN-γ levels (Fig. 6a; P < 0.05) and NK cell cytotoxicity (Fig. 6b; P < 0.05) were accelerated in NK-92 activated with IL-2, however, miR-544 overexpression diminished the levels of IFN-γ (Fig. 6a; P < 0.05) and cytotoxicity as compared to the IL-2 + pre-NC group (Fig. 6b; P < 0.05). Conversely, co-transfection of miR-544 with pcDNA-RUNX3 restored the function of NK cells on immune escape. This finding revealed that miR-544 played an important role in motivating immune escape in liver cancer cells.

### MiR-544 accelerated tumor growth and immune escape in a mouse model of liver cancer

**Fig. 7:**
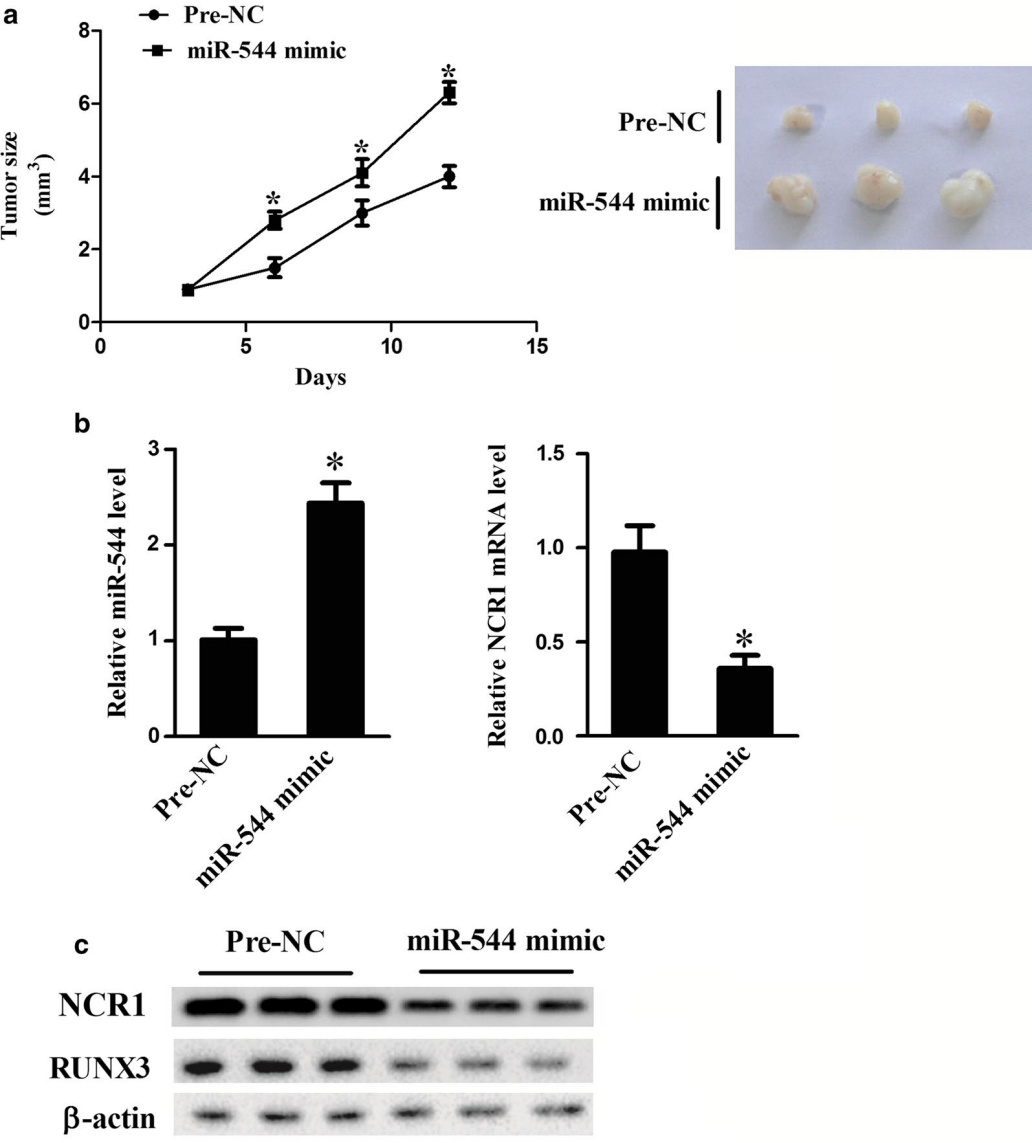
Effect of miR-544 on liver tumor immune escape in vivo. **a** The tumor volumes of mice model of liver cancer intravenously injected by LNK cells following transfection with pre-NC or miR-544 mimic. **b** The relative mRNA expression of miR-544 and NCR1 in mice model of liver cancer intravenously injected by LNK cells following transfection with pre-NC or miR-544 mimic. **c** The protein expression levels of NCR1 and RUNX3 in mice model of liver cancer intravenously injected by LNK cells following transfection with pre-NC or miR-544 mimic. *Refers to compare with the pre-NC group, P < 0.05

To further verify the role for miR-544 in immune escape against liver cancer cells in vivo, activated mouse LNK cells following transfection with pre-NC or miR-544 mimic were injected intravenously into mice model of liver cancer. LNK cells transfected with miR-544 mimic significantly led to enlargement of tumor volumes (Fig. 7a; P < 0.05), the enhancement of miR-544 and decrease of NCR1 expression at mRNA levels (Fig. 7b; P < 0.05) and the reduction of NCR1 and RUNX3 protein expression (Fig. 7c; P < 0.05). Collectively, these results manifested that miR-544 promoted immune escape in liver cancer by downregulating NCR1 via targeting RUNX3.

## Discussion

Tumor cells occasionally generated could be recognized as foreign bodies and killed by immune cells, however, tumor immune escape contributed to the occurrence, development and metastasis of malignant cells under the body’s immune surveillance, suggesting immune system played a vital role in tumorigenesis. In 2002, Schreiber et al. firstly proposed the concept of cancer immunoediting incorporating three phases: immune elimination, equilibrium and escape [22]. Tumor cell immune escape mechanisms mainly consisted of the modification of surface antigen or increased resistance to the cytotoxic effects of immune cells, such as NK cells.

NK cells, as primary effector cells in the innate immune system, are enriched in liver with organ specificity in terms of immune phenotype and function [23]. Growing evidence manifested that the altered expression of tumor-associated antigens along with the abnormal secretion of cytokines containing transforming growth factor-β (TGF-β) and IL in the tumor microenvironment disturbed the balance of NK cell inhibitory and activating receptors such as killer-immunoglobulin-like receptors (KIRs) and NCRs which greatly affected the cytotoxicity activity of NK cells, leading to the attenuation of anti-infection and anti-tumor responses [24]. In addition, RUNX3 was deemed to be a nuclear regulator in IL-dependent NK cell activation and thereby participated in tumor immune escape mechanism [25].

In this study, the increased expression levels of miR-544 was shown to be inversely associated with the decreased expression of RUNX3 and NCR1 in NK cells separated from patients with liver cancer, while miR-544 was downregulated in IL-2 activated-NK cells in vitro. Besides, Qiu et al. have found that miR-544 directly targeted RUNX3 in T helper cell immune response, and the latter was a transcription regulator of NCR1 [16, 20]. Thus, we speculated that miR-544 was closely related with RUNX3 and NCR1 expression in tumor immune escape. In order to verify this hypothesis, NK-92 cells were transfected with miR-544 inhibitor/mimic or their corresponding negative control sequence and the relative expression of NCR1 and RUNX3 at mRNA and protein levels were measured using qRT-PCR and western blotting. Results indicated that miR-544 knockdown significantly upregulated RUNX3, while miR-544 overexpression had an opposite effect. Furthermore, NK cells transfected with miR-544 mimic led to the decreased expression of NCR1, however, simultaneous overexpression of miR-544 and RUNX3 expedited NCR1 expression. These findings further elucidated that miR-544 directly targeted RUNX3 and sequentially regulated NCR1 in NK cells.

There has been increasing evidence for the vital roles of the intricate interaction between NK cell-activating receptors NKp46, NKp30 and NKp44 with tumor cell ligands in tumor immune escape [26]. For example, Trinidad et al. found that NKp30 and NKp46 downregulation were correlated with low cytotoxicity activity of NK cells in cervical cancer [27]. In this study, miR-544 overexpression depressed the cytotoxicity of activated NK cells and the secretion of IFN-γ as well as the percent of NKp46^+^NK cells, on the contrary, synchronous overexpression of miR-544 and RUNX3 reversed the trends, revealing that miR-544 alleviated the cytotoxicity of NK cells, weakened the lethal ability of NK cells against liver cancer cells and sequentially mediated immune escape.

Eventually, we established mice xenograft model of liver cancer to verify the role of miR-544 in tumor growth and immune escape. Results displayed that miR-544 overexpression in vivo promoted tumor growth, downregulated NCR1 and reduced RUNX3 expression.

## Conclusion

Overall, our study indicated that miR-544 overexpression in activated NK cells negatively regulated NCR1 through directly targeting RUNX3, resulting in suppressive cytotoxicity against liver cancer cells. Therefore, miR-544-induced NCR1 silencing was a promising factor for the immune escape of liver cancer.

## Abbreviations

NK: natural killer
IL: interleukin
NCR1: natural cytotoxicity receptor 1
qRT-PCR: quantitative real-time PCR

## References

[1] Ruiz-García AB, Olmos A (2017). First report of Grapevine Pinot gris virus in grapevine in Spain. Plant Dis.

[2] Gurusamy KS, Tsochatzis E, Thorburn D, Davidson B (2017). Management of people with early or very early stage hepatocellular carcinoma: a network meta-analysis. Cochrane Database Syst Rev.

[3] Wang EA, Stein JP, Bellavia RJ, Broadwell SR (2017). Treatment options for unresectable HCC with a focus on SIRT with Yttrium-90 resin microspheres. Int J Clin Pract..

[4] Tang ZY (2017). My view on the biological features and surgical treatment of liver cancer.

[5] Song P, Wang M, Ning WU, Liu F, Duan F, Yan J (2017). Interventional therapy for primary hepatic carcinoma associated with IVC-RA tumor thrombus: initial experience in 17 cases. J Interv Radiol..

[6] Honda Y, Kimura T, Aikata H, Nakahara T, Naeshiro N, Tanaka M, Miyaki D, Nagaoki Y, Kawaoka T, Takaki S (2014). Pilot study of stereotactic body radiation therapy combined with transcatheter arterial chemoembolization for small hepatocellular carcinoma. Hepatogastroenterology.

[7] Gwiasda J, Schrem H, Klempnauer J, Kaltenborn A (2017). Identifying independent risk factors for graft loss after primary liver transplantation. Langenbecks Arch Surg..

[8] Sharma P, Hu-Lieskovan S, Wargo JA, Ribas A (2017). Primary, adaptive, and acquired resistance to cancer immunotherapy. Cell.

[9] Aerts M, Benteyn D, Van VH, Thielemans K, Reynaert H (2016). Current status and perspectives of immune-based therapies for hepatocellular carcinoma. World J Gastroenterol.

[10] Beatty GL, Gladney WL (2015). Immune escape mechanisms as a guide for cancer immunotherapy. Clin Cancer Res.

[11] Guillerey C, Huntington ND, Smyth MJ (2016). Targeting natural killer cells in cancer immunotherapy. Nat Immunol.

[12] Sun C, Sun H, Xiao W, Zhang C, Tian Z (2015). Natural killer cell dysfunction in hepatocellular carcinoma and NK cell-based immunotherapy. Acta Pharmacol Sin.

[13] Halfteck GG, Elboim M, Gur C, Achdout H, Ghadially H, Mandelboim O (2009). Enhanced in vivo growth of lymphoma tumors in the absence of the NK-activating receptor NKp46/NCR1. J Immunol.

[14] Gras Navarro A, Bjorklund AT, Chekenya M (2015). Therapeutic potential and challenges of natural killer cells in treatment of solid tumors. Front Immunol.

[15] Sun C, Sun H, Zhang C, Tian Z (2015). NK cell receptor imbalance and NK cell dysfunction in HBV infection and hepatocellular carcinoma. Cell Mol Immunol.

[16] Lai CB, Mager DL (2012). Role of runt-related transcription factor 3 (RUNX3) in transcription regulation of natural cytotoxicity receptor 1 (NCR1/NKp46), an activating natural killer (NK) cell receptor. J Biol Chem.

[17] Mundybosse BL, Scoville SD, Li C, Mcconnell K, Mao HC, Ahmed EH, Zorko N, Harvey S, Cole J, Zhang X (2016). MicroRNA-29b mediates altered innate immune development in acute leukemia. J Clin Investig.

[18] Sullivan RP, Leong JW, Schneider SE, Ireland AR, Berrien-Elliott MM, Singh A, Schappe T, Jewell BA, Sexl V, Fehniger TA (2015). MicroRNA-15/16 antagonizes Myb to control NK cell maturation. J Immunol.

[19] Cioffi M, Trabulo SM, Vallespinos M, Raj D, Kheir TB, Lin ML, Begum J, Baker AM, Amgheib A, Saif J (2017). The miR-25-93-106b cluster regulates tumor metastasis and immune evasion via modulation of CXCL12 and PD-L1. Oncotarget.

[20] Qiu YY, Zhang YW, Qian XF, Bian T (2017). miR-371, miR-138, miR-544, miR-145, and miR-214 could modulate Th1/Th2 balance in asthma through the combinatorial regulation of Runx3. Am J Transl Res.

[21] Cheng M, Zhi K, Gao X, He B, Li Y, Han J, Zhang Z, Wu Y (2013). Activation of cellular immunity and marked inhibition of liver cancer in a mouse model following gene therapy and tumor expression of GM-SCF, IL-21, and Rae-1. Mol Cancer.

[22] Dunn GP, Bruce AT, Ikeda H, Old LJ, Schreiber RD (2002). Cancer immunoediting: from immunosurveillance to tumor escape. Nat Immunol.

[23] Male V (2017). Liver-resident NK cells: the human factor. Trends Immunol.

[24] Childs WR, Pantin JM (2017). NK cells.

[25] Levanon D, Negreanu V, Lotem J, Bone KR, Brenner O, Leshkowitz D, Groner Y (2014). Transcription factor Runx3 regulates interleukin-15-dependent natural killer cell activation. Mol Cell Biol.

[26] Moretta L, Bottino C, Pende D, Castriconi R, Mingari MC, Moretta A (2006). Surface NK receptors and their ligands on tumor cells. Semin Immunol.

[27] Garcia-Iglesias T, Del Toro-Arreola A, Albarran-Somoza B, Del Toro-Arreola S, Sanchez-Hernandez PE, Ramirez-Duenas MG, Balderas-Pena LM, Bravo-Cuellar A, Ortiz-Lazareno PC, Daneri-Navarro A (2009). Low NKp30, NKp46 and NKG2D expression and reduced cytotoxic activity on NK cells in cervical cancer and precursor lesions. BMC Cancer.

